# Effect of Melatonin on Fruit Quality via Decay Inhibition and Enhancement of Antioxidative Enzyme Activities and Genes Expression of Two Mango Cultivars during Cold Storage

**DOI:** 10.3390/foods11203209

**Published:** 2022-10-14

**Authors:** Alagie Njie, Wen’e Zhang, Xiaoqing Dong, Chengyu Lu, Xuejun Pan, Qingguo Liu

**Affiliations:** 1 College of Agriculture, Guizhou University, Guiyang 550025, China; 2 School of Agriculture and Environmental Sciences, University of The Gambia, Kanifing P.O. Box 3530, The Gambia; 3 Institute of Subtropical Crops, Guizhou Academy of Agricultural Sciences, Fenglindong Road, Xingyi, Guiyang 562400, China

**Keywords:** mango fruit, melatonin treatment, cold storage, decay, physiological and metabolic processes

## Abstract

The postharvest deterioration of mango fruits is a critical issue limiting mango storage and preservation due to its climacteric nature. This study evaluated the storage behavior of two mango cultivars and their response to exogenous melatonin (MT, 1000 μmol L^−1^) treatment in attenuating fruit decay and enhancing fruits’ physiological and metabolic processes and gene relative expression subjected to cold storage. MT treatment in both mango cultivars significantly delayed weight loss, firmness, respiration rate, and decay incidence. However, MT did not influence the TSS, TA, and TSS:TA ratio regardless of the cultivar. Moreover, MT inhibited the decrease in total phenol and flavonoid content and AsA content while delaying the increase in the MDA content of mango during storage in both cultivars. In addition, MT dramatically inhibited the enzyme activity of PPO. In contrast, an increase in the activities of antioxidant enzymes (SOD and APX) and PAL and their genes’ relative expression was noticed in MT-treated fruits versus control in both cultivars. However, MT treatment was cultivar dependent in most parameters under study. These results demonstrated that MT treatment could be an essential postharvest treatment in minimizing decay, maintaining fruit quality, and extending mango fruits’ postharvest shelf life by enhancing the physiological and metabolic processes during cold storage.

## 1. Introduction

Mango (*Mangifera indica*) is one of the most vital tropical fruits globally due to its unique features: its pleasant flavor, attractive color, distinct taste and aroma, and rich nutritional value (carbohydrates, minerals, fibers, vitamins, polyphenols) [1,2]. In addition, mango fruits are categorized as a climacteric type with relatively high respiration. Mangoes are usually harvested in July and August at very high temperatures, and the harvested mango fruits ripen within a few days under ambient conditions. These factors lead to a relatively short postharvest shelf life due to rapid fruit decay and deterioration, a limiting factor in the long-distance transport and marketing of the fruit [1,3].

Most fruits, including mango, that contain very high water content are highly vulnerable to the attack of microbes, which ultimately cause decay in the harvested produce and reduce the shelf life and quality. Therefore, it is crucial to protect them from postharvest decay caused by microbes, bruises, and mechanical damage while improving shelf life and maintaining postharvest quality. Melatonin (N-acetyl-5-methoxytryptamine, MT), a derivative of tryptophan, is a vital indoleamine hormone widely found in various plant species, including apple, strawberry, grape, cherry, banana, kiwi, etc. [4]. MT plays a role in several physiological processes, such as regulating plant growth and development, delaying leaf senescence, and promoting fruit ripening [5,6]. MT, a potent antioxidant, improves the resistance of plants to both abiotic and biotic stresses by eliminating reactive oxygen species (ROS), elevating the antioxidant system, and increasing the efficiency of other antioxidants in plants. MT has shown its effectiveness in controlling decay by suppressing the activities of various fungi, bacteria, and other microbes, and also boosts the physiological performance of fruits against unfavorable conditions [7]. Similarly, MT was also reported to scavenge free radicals, ROS, and reactive nitrogen species (RNS) directly, act as a signaling molecule at the cellular level, and increase the number of antioxidant enzymes, which promotes its activity as an antioxidant [8]. Among the leading causes of fruit decay during storage is the development of rots caused by a range of fungi. Total phenolic content and antioxidant activity were more significant in MT-treated Santa Rosa plums than in the control, substantially reducing the decay rate during cold storage [9].

Recently, exogenous MT treatment has been tested as an effective postharvest treatment in different fruits. Some researchers found that MT was effective in promoting postharvest banana ripening [10], reducing senescence, and improving the quality of peach fruit and strawberry, respectively [11,12], minimizing chilling injury in peach fruit [13]. Rastegar et al. [14] found that MT at 1000 μmol L^−1^ maintained fruit firmness, ascorbic acid content (AsA), the content of phenolic compounds, and the antioxidant capacity of mango during storage. Similar research by Liu et al. [15] stated that the application of MT (0.5 mM, immersion for 1 h) to ‘Guifei’ mangoes effectively delayed the changes in ripening parameters, including firmness, total soluble solids content (TSS), titratable acidity (TA), and respiration rate. Moreover, MT significantly controlled polyphenol oxidase (PPO) activity and increased the activity of the catalase (CAT) and peroxidase (POD) enzymes during storage [14]. However, a more recent paper by Bhardwaj et al. [16] discovered that MT treatment has a different effect on different cultivars of mango fruits. They found that ‘Langra’ mangos responded best to MT treatment by increasing their chilling tolerance during storage, while ‘Gulab Jamun’ mangos did not show any significant effect [16], which demonstrates that the MT effect is cultivar dependent. MT upregulates critical genes’ (*PpSODs*, *PpCATS*, *PpAPXs*, and *PpGRs*) expression in peaches’ antioxidant defense [17]. In addition, the transcript expression of phenolic biosynthesis genes was upregulated by MT treatment, indicating that MT could activate the phenylpropanoid pathway [18]. MT reduces ethylene production by regulating the expression of the *MaACO* and *MaACS* genes and delays sharp changes in quality indices in bananas [19]. Furthermore, exogenous MT significantly regulated the transcript levels of the expression of critical genes involved in antioxidative defense (*SOD*, *CAT*, *APX*, *POD*) and *PAL* resulting in increased tolerance to drought stress in Chinese hickory plants [20].

‘Guiqi’ and ‘Tainong 1′ used in this research are two of Guizhou province’s most cultivated mango cultivars. ‘Guiqi’, also known as ‘Gui re 82′, is a mango cultivar with a thin peel and is rich in aroma content, TSS, and TA [21]. ‘Tainong 1′, an early maturing cultivar with a solid pine flavor, is rich in polyphenols and flavonoids and has high antioxidant scavenging inhibition [22]. Zhang et al. [23] discovered that hot water treatment at 55 °C for 10 min effectively enhanced the chilling tolerance in ‘Tainong 1′ mango fruit during ambient storage after exposure to low temperature [23]. The above two cultivars have different physiological characteristics. However, it is speculated that the storage characteristics of these two cultivars, especially ‘Guiqi’, are not evident. Therefore, the current study aims to compare the storage characteristics of two mango cultivars after MT treatment subjected to cold storage. The study also aims to evaluate the effect of exogenous MT treatment on extending the shelf life of these two cultivars by attenuating fruit decay and enhancing fruits’ physiological and metabolic processes and genes relative expressions during storage. The results of this study will give an insight into the storage behavior of these two cultivars of mango and their response to MT treatment. It is also assumed that this research will give insight into how MT treatment will help minimize fruit deterioration and improve antioxidative processes and relative gene expression, thereby maintaining quality and extending the shelf life of mango fruits.

## 2. Materials and Methods

### 2.1. Plant Materials

Mango fruits of the early maturing ‘Tainong 1′ and late-maturing ‘Guiqi’ were harvested from Guizhou Mountain Mango Base farm in Wangmo county, Guizhou province (Latitude 25°6′ North, and Longitude 106°6′ East), on 21 July and 8 August, respectively. In total, 240 fruits of ‘Tainong 1′ and ‘Guiqi’ each were harvested at the physiological green mature stage and packed in cardboard boxes. The fruit of each layer was separated with soft fabric to avoid compression injury. After sorting to choose fruits of uniform weight with no visible blemishes, they were transported to the laboratory within 3 h using cardboard boxes via a van with air conditioning at 25 ± 1 °C and 50–60 ± 0.5% relative humidity (RH). Upon arrival at the laboratory, the harvested fruits were treated using sodium hypochlorite (5% *v*/*v*) by immersing them for 10 min. Then, they were rinsed using distilled water and air-dried.

### 2.2. Melatonin Treatment

A complete randomized design (CRD) with three replications was used in this research. Each treatment was assigned 56 fruits and replicated three times. According to our pre-experiment, 1000 μmol L^−1^ MT was used as the final concentration in which fruits were immersed for 30 min. Treatment using distilled water for 30 min served as the control. The fruits were then air-dried at room temperature and stored in a refrigerator at 13 ± 1 °C and 80 ± 1% RH. The fruits were evaluated at three-day intervals during a one-month storage period.

### 2.3. Quality Parameters

#### 2.3.1. Weight Loss

The fruit weight loss was measured using an electronic scale and measured in grams. The weight loss was measured every three days using 20 fruits from each treatment, compared with the initial weight, and expressed in percentage.

$$weight\;loss\;\left(\%\right)=\frac{initial\;weight-final\;weight}{\;initial\;weight}\times 100$$

#### 2.3.2. Firmness

A handheld penetrometer (GY-4, Yueqing Edberg Instrument Co., Ltd., Yueqing, China) was used to determine the firmness of the mango fruits (*n* = 3). A peel from the mango fruit was removed from both ends, and the firmness was measured in the equatorial region using a 3.5 mm probe. Firmness was expressed as a force in Newton (N).

#### 2.3.3. Decay Incidence Rate

Decay incidence of mango fruits was the number of fruits showing decay symptoms (rot, lesions, or visible fungal growth) relative to the total number of fruits and expressed in a percentage (percentage). Twenty fruits from each treatment were used to measure decay incidence, and a visual inspection was carried out every three days.

$$Decay\;incidence\;\left(\%\right)=\frac{number\;of\;fruits\;showing\;decay\;symptoms}{\;total\;number\;of\;fruits}\times 100$$

#### 2.3.4. Respiration Rate

The respiration rate expressed as mg CO_2_·kg^−1^·h^−1^ FW was determined as modifications described by [24]. Six (6) fruits of each treatment were enclosed in a 9 L glass jar at room temperature for 30 min, and three biological replicates were performed. The respiration rate was calculated by the concentration of CO_2_, which was measured by a CO_2_ gas analyzer (Telaire-7001, Goleta, CA, USA).

### 2.4. Nutritional Parameters

#### 2.4.1. Total Soluble Solid Content (TSS), Titratable Acidity (TA), and TSS:TA

TSS was measured using a refractometer (Hybrid, PAL-BX Ι ACID 1, ATAGO, TOKYO) at room temperature and expressed as Brix percentage. For the measurement of TA, 20 μL of mango juice was pipetted with 980 μL of distilled water, and the concentration of TA was determined using a refractometer. The results were expressed in percentage (%). The ripening index was calculated as the ratio of TSS:TA according to [25].

#### 2.4.2. Ascorbic Acid (AsA) Content

In total, 0.5 g of pulp sample was weighed and homogenized with 2 mL of 50 g/L trichloroacetic acid (TCA) in a mortar and grounded in ice condition. The homogenate was centrifuged at 12,000 rpm for 20 min, and the supernatant was collected and used for the assay of the AsA content. Briefly, 1 mL of the sample extract was mixed with 1 mL of 50 g/L TCA solution, and 1 mL of anhydrous alcohol was added and mixed well by vortex. Afterward, 0.5 mL of 4% phosphoric acid was added and vortexed again. After 5 min, 1 mL of 5 g/L BP was added and mixed well, and 0.5 mL of 0.3 g/L FeCl_3_ was then added and mixed well with the vortex. The mixed solution was placed in the dark at 30 °C for 60 min, and the absorbance was recorded at 534 nm against the blank. Ascorbic acid was used as the standard, and the AsA content was expressed in a 100 g sample (fresh weight), i.e., mg·100 g^−1^ FW.

#### 2.4.3. Total Phenol and Flavonoid Content

One gram (1 g) of frozen mango samples was ground using a mortar and pestle and extracted in 5 mL of 95% (*v*/*v*) methanol. The homogenate was sonicated for 30 min, followed by centrifuging at 11,000× *g* for 20 min at 4 °C. Then, the supernatant was transferred to a new tube and stored at −20 °C, used to determine the total phenol and flavonoid content.

The total phenol content was assayed using the method of Liu et al. [12], with modifications. Briefly, 0.5 mL volume of the methanol extract was mixed with 1 mL Folin–Ciocalteu reagent, following which the reaction mixture was allowed to stand for 5 min at ambient temperature. Next, 3 mL of 7.5% sodium carbonate was added and left to react for 30 min. The absorbance was measured at 765 nm. The results were expressed as mg GAE·100 g^−1^ FW against gallic acid as a standard.

The total flavonoid content was determined using modifications by the colorimetric method [12]. Methanol extract (1 mL) was mixed with 5% NaNO_2_ solution (0.3 mL). After being allowed to stand for 5 min, the mixture was combined with a 10% AlCl_3_ solution (0.3 mL), while 1 M sodium hydroxide (NaOH) (1.5 mL) and distilled water (1.9 mL) were added after 5 min. The absorbance of the reaction mixture was recorded at 510 nm. Quercetin (QE) was used as a standard, and the results were expressed as mg QE·100 g^−1^ FW.

### 2.5. Malondialdehyde (MDA) Content

The MDA content was assayed according to the thiobarbituric acid method [26] with slight modification. Briefly, 1 g samples were ground as homogenate using 5 mL cold 10% (*w*/*v*) TCA solution and centrifuged at 10,000× *g* for 20 min at 4 °C. Then, 2 mL supernatant and 3 mL 0.67% (*w*/*v*) thiobarbituric acid (TBA) were mixed and heated for 20 min in a boiling water bath at 50 °C. After cooling, the absorbance of the supernatant was measured at 450, 532, and 600 nm. Finally, the MDA content was calculated using the formula and expressed as nmol·g^−1^ FW basis:

$$MDA\;content\;\left(nmol\cdot g^{-1}\right)=6.45*\left(OD_{532}-OD_{600}\right)-0.56*OD_{450}$$

### 2.6. Enzymes Activities

First, 0.5 g mango samples were rapidly homogenized on the ice with 8 mL ice-cold sodium phosphate buffer (PBS, 50 mmol L^−1^, pH 7.8) containing 1% (*w*/*v*) polyvinyl polypyridine (PVP). Then, the homogenate was centrifuged at 12,000× *g* for 30 min at 4 °C, following which the supernatant was divided into 1.5 mL centrifuge tubes and stored at −70 °C as a crude extract for PPO, SOD, and APX determination.

#### 2.6.1. Polyphenol Oxidase (PPO) Activity

A 4.0 mL of 50 μmol L^−1^ sodium acetate buffer, pH 5.5, was mixed with 0.5 mL of 50 mmol L^−1^ catechol solution, and finally, 100 μL of enzyme extraction solution was added and started timing immediately. The absorbance of the reaction system at 420 nm was recorded at 15 s as an initial value and then recorded at 1 min intervals for 6 min. The PPO enzyme activity was expressed in U·min^−1^·g^−1^ FW.

#### 2.6.2. Phenylalanine Ammonia-Lyase (PAL) Activity

PAL activity was assayed using a plant PAL kit (A137-1-1, Nanjing Jiancheng Bioengineering Institute, Nanjing, China) following the manufacturer’s instructions and absorbance measurement at 290 nm and expressed in U·min^−1^·g^−1^ FW.

#### 2.6.3. Superoxide Dismutase (SOD) Activity

The reaction mixture includes 1.7 mL of 50 μmol L^−1^ sodium phosphate buffer, pH 7.8, 0.3 mL of 130 μmol L^−1^ methionine solution, 0.3 mL of 750 μmol L^−1^ NBT solution, 0.3 mL of 100 μmol L^−1^ EDTA-Na_2_ solution, 0.3 mL of 20 μmol L^−1^ riboflavin solution, and 0.1 mL of enzyme extract. The reaction mixture was exposed under a 4000 lx fluorescent lamp for 10 min before measuring the SOD activity at 560 nm. The reaction system’s inhibition of the photochemical reduction of NBT was expressed as one SOD activity unit (U) per min per gram of fresh weight at 50%.

#### 2.6.4. Ascorbate Peroxidase (APX) Activity

A 2.6 mL reaction buffer was added to 0.1 mL of enzyme extract in a test tube. Then, 3 mL of 2 μmol L^−1^ H_2_O_2_ solution was added to initiate the enzymatic reaction and immediately mixed. The absorbance at 290 nm of the initial reaction was recorded from 15 s after then at 30 s interval for 3 min. The APX enzyme activity was expressed in U·min^−1^·g^−1^ FW.

### 2.7. RNA Isolation and Gene Expression Analysis

Total RNA was extracted from frozen mango pulp for gene expression analysis using RNAprep Pure kit (TIANGEN BIOTECH (BEIJING) CO., LTD DP140916, Beijing, China) and further assessed for quantity and quality with a NanoDrop One. Then, 2 μg of extracted RNA were further treated with FastKing-RT SuperMix and further converted into cDNA with the FastKing gDNA Dispelling RT SuperMix according to the manufacturer’s instruction (TIANGEN BIOTECH (BEIJING) CO., LTD; KR170801). Specific primers of *PPO*, *PAL*, *MnSOD*, and *APX* were obtained from National Center for Biotechnology Information (NCBI) and designed by their nucleotide sequence using PRIMER 6 software, and then synthesized by a biological company (Tsingke Biotech (Guiyang) Co., Ltd., Guiyang, China). The qRT-PCR analysis of *PPO*, *PAL MnSOD*, and *APX* genes in mango was performed using gene-specific primers (Table 1) in a total volume of 20 μL with Bioer Real-Time PCR Detection Systems (BIOER Technology Ltd., Gene-9660, Hangzhou, China) using TB Green Premix EX Taq II (TAKARA BIO INC); Cat. RR820A, Beijing, China). The Real-Time qPCR reaction was performed with the following thermal profile: 95 °C 30 s, 40 cycles of 95 °C 5 s and 60 °C 34 s, and finishing with a final cycle at 95 °C 15 s, 60 °C 60 s, and 95 °C per 15 s, to define the reaction melting curve. The qPCR data were calibrated relative to *Actin* as a reference gene at zero time for each treatment, following the 2^−ΔΔCt^ method for relative quantification. The values represent the mean of three biological replicates.

### 2.8. Statistical Analysis

Microsoft Excel (V. 2010, Washington, DC, USA), OriginPro (V. 2022b, Northampton, MA, USA), and SPSS (V.26.0, New York, NY, USA) were used as the statistical software to analyze the results obtained from the experiment. ANOVA analysis was used to compare the mean difference. *p* < 0.05 was used as the statistical difference between means using Tukey’s test. The storage period, cultivar, and treatment were used as the source of variations in this research.

## 3. Results

### 3.1. Effects of MT on Quality Parameters

#### 3.1.1. Weight Loss and Firmness

**Figure 1:**
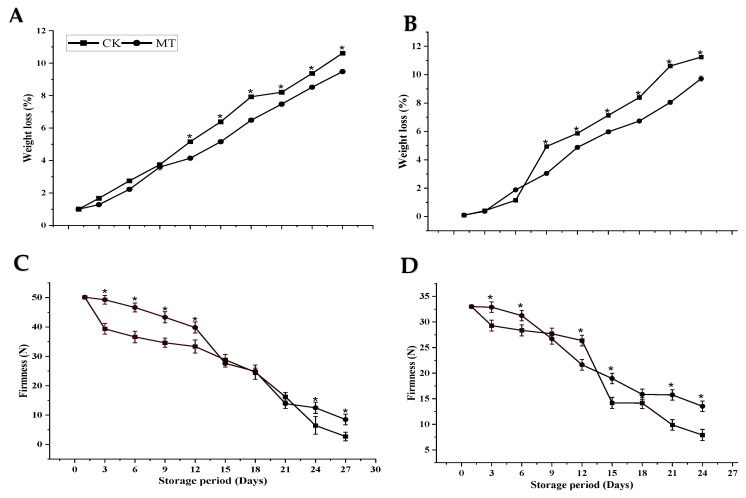
Weight loss (**A**,**B**) and firmness (**C**,**D**) of ‘Guiqi’ (**A**,**C**) and ‘Tainong 1′ (**B**,**D**) fruits treated with 1000 μmol L^−1^ MT stored at 13 ± 1 °C and 80 ± 1% RH. Each value is the mean for three replicates, and vertical bars indicate the standard error (SE). Error bars with an asterisk (*) on the same storage period show a significant difference between the treatments (*p* < 0.05).

During the storage period, weight loss measured in percentage increased steadily in both cultivars regardless of the treatment (Figure 1A,B). However, at the end of the storage period, MT treatment illustrated a lower mean percentage loss in weight than control fruits in both cultivars (Figure 1). The lowest weight loss for MT treatment was 9.48% for ‘Guiqi’ and 9.72% for ‘Tainong 1′ as compared to the control with 10.62% for ‘Guiqi’ and 11.24% for ‘Tainong 1′ at 24 d and 27 d storage periods, respectively. There was no cultivar difference in terms of weight loss. The difference between MT and control in both cultivars was significant (*p* < 0.05).

The firmness of both cultivars showed a constant decreasing trend, which signifies that the fruits became softer with an increased storage period (Figure 1C,D). However, at the end of the storage period, the MT-treated fruits maintained higher firmness than the control fruits with a significant difference (*p* < 0.05). MT treatment delayed fruit firmness in ‘Guiqi’ almost thrice that of the control at 27 d (8.47 ± 1.83 and 2.72 ± 1.47), respectively, while in ‘Tainong 1′, the delay difference between MT and control at 24 d was almost twice (13.52 ± 1.03 and 7.91 ± 1.09), respectively.

#### 3.1.2. Decay Incidence and Respiration Rate

**Figure 2:**
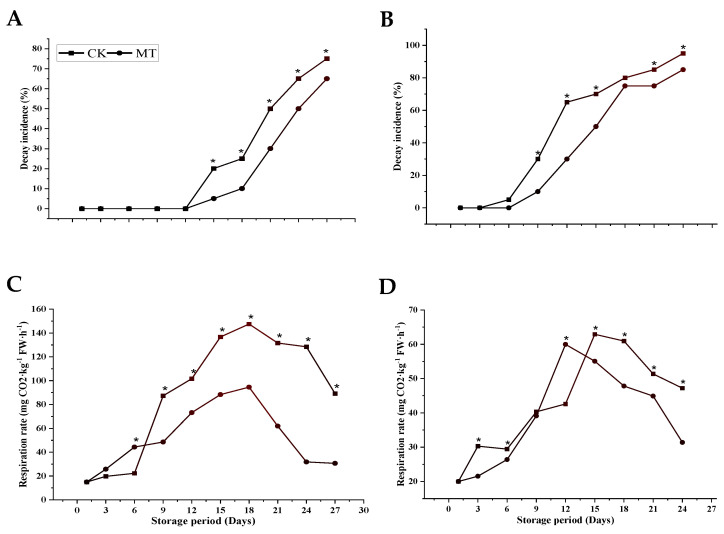
Decay incidence (**A**,**B**) and respiration rate (**C**,**D**) of ‘Guiqi’ (**A**,**C**) and ‘Tainong 1’ (**B**,**D**) fruits treated with 1000 μmol L^−1^ MT. Each value is the mean for three replicates, and vertical bars indicate the standard error (SE). Error bars with an asterisk (*) on the same storage period show a significant difference between the treatments (*p* < 0.05).

Fruits started to show signs of decay incidence after 12 d in the ‘Guiqi’ cultivar and 3 d in ‘Tainong 1′, and the trend continued to increase until the end of the storage period (Figure 2A,B). MT treatment showed a lower incidence of decay compared to the control treatment in both cultivars with a statistically significant difference at (*p* < 0.05). The highest decay percentages were 65% and 85% for MT treatment in ‘Guiqi’ and ‘Tainong 1′, and 75% and 95% for the control, respectively, for 24 d and 27 d storage periods, respectively.

Both treatments showed a similar respiration pattern during storage in both cultivars (Figure 2C,D). However, from 9 d to 27 d, MT-treated fruits showed a much lower respiration rate than the control in ‘Guiqi’, with a significant difference (*p* < 0.05). While in ‘Tainong 1′, the difference was significant from 12 d to 24 d, in which MT-treated fruits showed lower respiration at most stages of the storage period. The respiration peak in MT-treated fruits was observed on 18 d (94.56 ± 0.05 mg CO_2_·kg^−1^ FW·h^−1^) in ‘Guiqi’, while in ‘Tainong 1′, it was observed on 12 d (59.96 ± 0.06 mg CO_2_·kg^−1^ FW·h^−1^) as compared to the control with a peak at (147.49 ± 0.14 mg CO_2_·kg^−1^ FW·h^−1^, and 62.91 ± 0.05 mg CO_2_·kg^−1^ FW·h^−1^) in ‘Guiqi’ and ‘Tainong 1′, respectively.

### 3.2. Effect of MT on Nutritional Parameters of Mango

#### 3.2.1. TSS, TA, and TSS:TA

**Figure 3:**
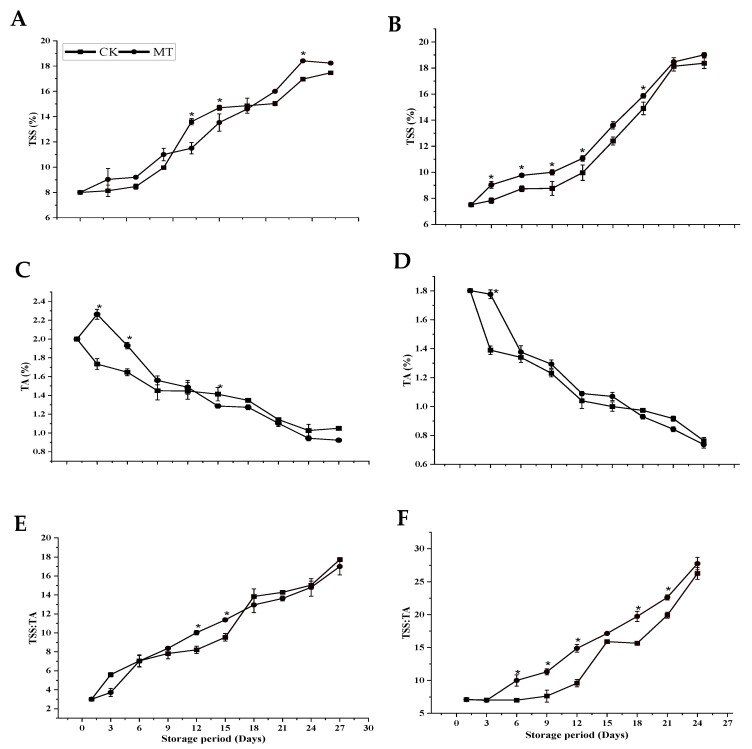
TSS (**A**,**B**), TA (**C**,**D**), and TSS:TA ratio (**E**,**F**) of ‘Guiqi’ (**A**,**C**,**E**) and ‘Tainong 1’ (**B**,**D**,**F**) fruits treated with 1000 μmol L^−1^ MT. Each value is the mean for three replicates, and vertical bars indicate the standard error (SE). Error bars with an asterisk (*) on the same storage period show a significant difference between the treatments (*p* < 0.05).

MT treatment did not affect TSS content in this study due to its higher concentration than the control. At the end of the storage period, the TSS content in MT was 18.23% and 19.01% for ‘Guiqi’ and ‘Tainong 1’, respectively. While in control, 17.47% for ‘Guiqi’ and 18.37% for ‘Tainong 1’ were observed. However, the difference was not significant (*p* < 0.05) between the treatments in each cultivar at the end of the storage period (Figure 3A,B). TA showed a decreasing pattern in both cultivars regardless of the treatment throughout the storage period (Figure 3C,D). From 6 d in ‘Guiqi’ and 9 d in ‘Tainong 1’, MT-treated fruits did not significantly affect the TA content in both cultivars. At the end of the storage period, MT treatment showed a lower TA content (0.92% and 0.74% for ‘Guiqi’ and ‘Tainong 1’, respectively) than the control (1.05% and 0.76% for ‘Guiqi’ and ‘Tainong 1’, respectively), but the difference was not statistically significant (*p* < 0.5). A continuous increase in the TSS:TA ratio in both cultivars was observed, indicating increasing maturity and ripening during storage. At the end of the storage period, MT treatment did not influence the TSS:TA ratio reduction in both cultivars (Figure 3E,F). However, there was a lower TSS:TA ratio by MT in ‘Guiqi’ than in the control, while in ‘Tainong 1’, the TSS:TA ratio was higher for MT than in the control.

#### 3.2.2. AsA Content

**Figure 4:**
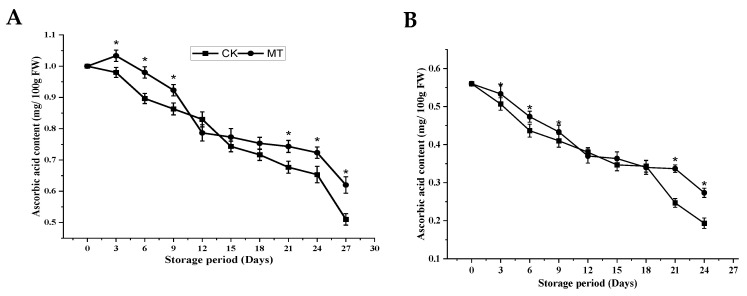
Ascorbic acid content (**A**,**B**) of ‘Guiqi’ (**A**) and ‘Tainong 1’ (**B**) fruits treated with 1000 μmol L^−1^ MT. Each value is the mean for three replicates, and vertical bars indicate the standard error (SE). Error bars with an asterisk (*) on the same storage period show a significant difference between the treatments (*p* < 0.05).

A continuous decrease in the AsA content was observed in both cultivars throughout the storage period regardless of the treatment (Figure 4A,B). However, MT treatment resulted in a higher AsA content than the control fruits at the end of the storage period, and the results were statistically significant (*p* < 0.05). At the 27 d, the AsA in ‘Guiqi’ was 0.62 ± 0.03 mg/100 g FW for MT treatment as compared to 0.51 ± 0.02 mg/100 g FW for the control, while at 24 d in ‘Tainong 1’, 0.27 ± 0.01 mg/100 g FW for MT was observed compared to 0.19 ± 0.14 mg/100 g FW for the control. Moreover, the interaction between the treatment and storage period was more significantly affected by MT treatment in ‘Guiqi’ than in ‘Tainong 1’ (*p* < 0.05) during most stages of the storage period.

#### 3.2.3. Total Flavonoid and Phenol Content

**Figure 5:**
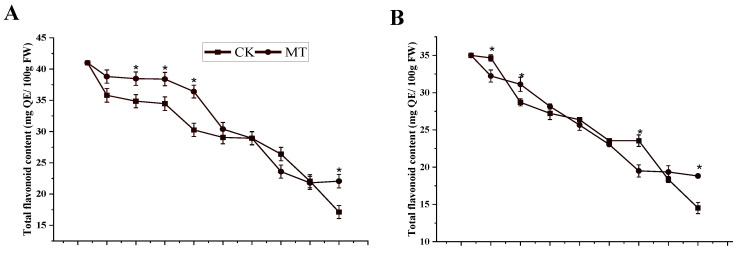

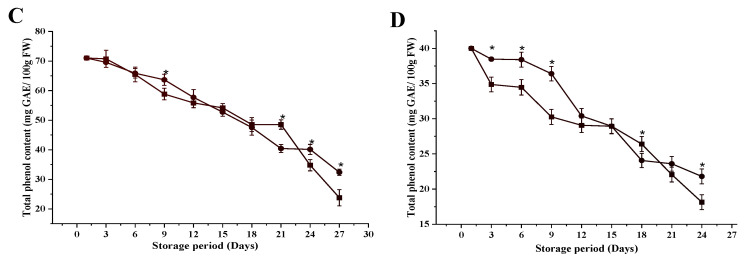
Total flavonoid content (**A**,**B**) and total phenol content (**C**,**D**) of ‘Guiqi’ (**A**,**C**) and ‘Tainong 1’ (**B**,**D**) fruits treated with 1000 μmol L^−1^ MT. Each value is the mean for three replicates, and vertical bars indicate the standard error (SE). Error bars with an asterisk (*) on the same storage period show a significant difference between the treatments (*p* < 0.05).

Figure 5A–D illustrated a decline in the mean values of the total flavonoid and phenol content in both the control fruits and the MT-treated fruits throughout the storage period, regardless of the cultivar. However, MT treatment showed higher significant values in the total flavonoid content at the end of the storage period than the control treatment (*p* < 0.05) in both cultivars. The lowest content of total flavonoid in the MT treatment was (22.07 ± 1.08 and 18.82 ± 0.26 mg QE/100 g FW) compared to the control (17.13 ± 1.03 and 14.52 ± 0.75 mg QE/100 g FW) in ‘Guiqi’ and ‘Tainong 1’, respectively. A similar trend was noticed in the total phenol content, in which the MT treatment showed higher mean values (32.38 ± 1.04 and 21.80 ± 1.06 mg GAE/100 g FW) than in the control (23.78 ± 2.76 and 18.13 ± 1.06 mg GAE/100 g FW) in ‘Guiqi’ and ‘Tainong 1’, respectively. The interaction between the treatment and the storage period was similar in both cultivars but slightly more pronounced in ‘Tainong 1’ than in ‘Guiqi’.

### 3.3. MDA Content

**Figure 6:**
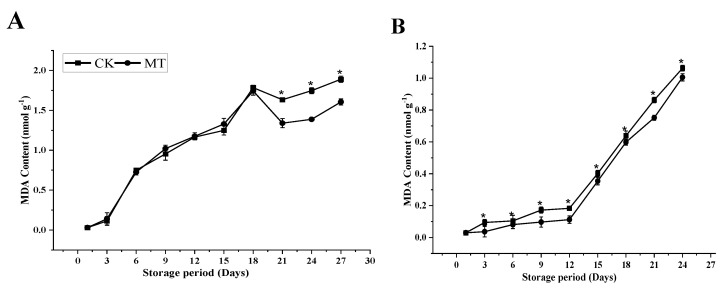
MDA content (**A**,**B**) of ‘Guiqi’ (**A**) and ‘Tainong 1’ (**B**) fruits treated with 1000 μmol L^−1^ MT. Each value is the mean for three replicates, and vertical bars indicate the standard error (SE). Error bars with an asterisk (*) on the same storage period show a significant difference between the treatments (*p* < 0.05).

MDA, the main product of membrane lipid peroxidation, was measured in both cultivars during the 27 d for ‘Guiqi’ and 24 d for the ‘Tainong 1’ storage periods (Figure 6A, B). In Guiqi, the MDA content increased significantly up to 18 d of storage in both the control and MT-treated fruit and then slightly declined on 21 d of storage, then increased for the rest of the storage period. However, lower MDA content was observed in the MT treatment (1.60 ± 0.04 nmol g^−1^) than in the control (1.89 ± 0.04 nmol g^−1^) on 27 d. In ‘Tainong 1’, MDA slightly increased until 15 d, where a massive increase occurred until the final day of the storage period. However, MDA was lower in the MT-treated fruit (1.01 ± 0.02 nmol g^−1^) than in the control fruit (1.06 ± 0.02 nmol g^−1^) on 24 d. The difference between MT and the control in MDA content inhibition was more pronounced in ‘Guiqi’ than in ‘Tainong 1’ at the final stage of storage.

### 3.4. Effect of MT on Enzyme Activities

#### 3.4.1. PPO and PAL Activity

**Figure 7:**
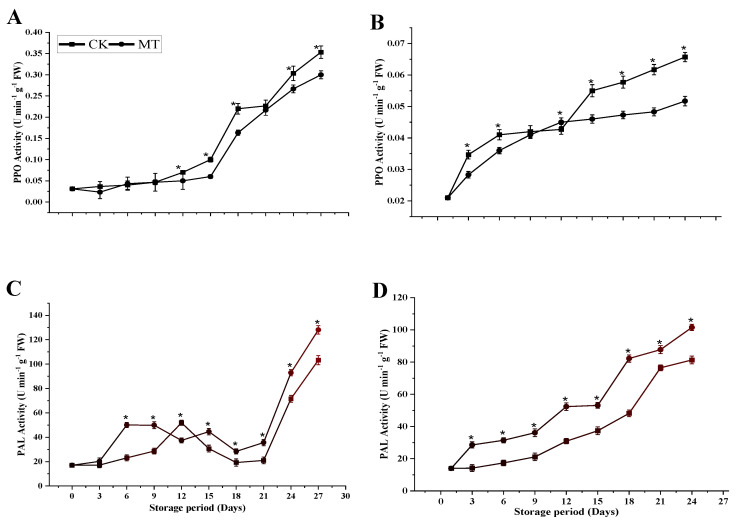
PPO activity (**A**,**B**) and PAL activity (**C**,**D**) of ‘Guiqi’ (**A**,**C**) and ‘Tainong 1’ (**B**,**D**) fruits treated with 1000 μmol L^−1^ MT. Each value is the mean for three replicates, and vertical bars indicate the standard error (SE). Error bars with an asterisk (*) on the same storage period show a significant difference between the treatments (*p* < 0.05).

Figure 7A,B manifested a gradually increased PPO activity during storage, reaching a maximum level at the end of storage in the control and MT-treated fruits in both cultivars. The PPO activity in the control samples was significantly higher (0.35 ± 0.01 and 0.07 U min^−1^ g^−1^ FW for ‘Guiqi’ and ‘Tainong 1’, respectively) than in the MT-treated fruits (0.3 ± 0.01 and 0.05 U min^−1^ g^−1^ FW for ‘Guiqi’ and ‘Tainong 1’, respectively) at the end of the storage (*p* < 0.05). However, the difference between MT and control treatments in response to the PPO activity was more pronounced in ‘Tainong 1’ than in ‘Guiqi’.

As depicted in Figure 7C,D, the PAL enzyme activity in control fruits and fruits treated with MT increased within the first 12 d of storage. It then decreased until 21 d, and then massively increased until 27 d in ‘Guiqi’, while in ‘Tainong 1’, there was an increasing trend throughout the storage period. At the end of the storage period, the fruits treated with MT exhibited significantly higher PAL enzyme activity during storage (*p* < 0.05) than the control treatment, regardless of the cultivar (Figure 7C,D). However, higher PAL activity by MT was more pronounced in ‘Tainong 1’ than in ‘Guiqi’ during storage.

#### 3.4.2. SOD and APX Activity

**Figure 8:**
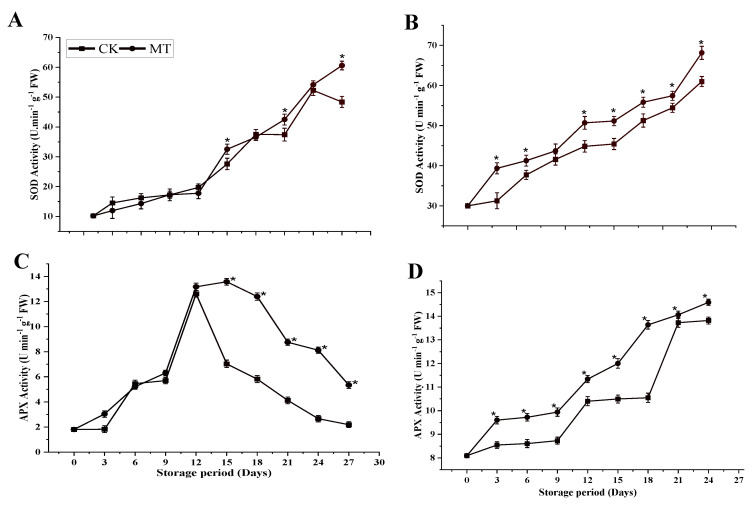
SOD activity (**A**,**B**) and APX activity (**C**,**D**) of ‘Guiqi’ (**A**,**C**) and ‘Tainong 1’ (**B**,**D**) fruits treated with 1000 μmol L^−1^ MT. Each value is the mean for three replicates, and vertical bars indicate the standard error (SE). Error bars with an asterisk (*) on the same storage period show a significant difference between the treatments (*p* < 0.05).

Figure 8A,B demonstrated that the SOD activity in both cultivars followed a similar trend during the storage period. MT treatment was 1.25 times higher than the control at 27 d in ‘Guiqi’, and 1.12 times higher at 24 d in ‘Tainong 1’. However, the effect of MT on the SOD activity was more pronounced in ‘Tainong 1’ than in ‘Guiqi’ since MT (*p* < 0.05) did not significantly influence the treatment effect and storage period in ‘Guiqi’.

As depicted in Figure 8C,D, there is a difference in the pattern of the APX activity between the two cultivars. The APX activity in ‘Guiqi’ increased in both treatments, where they reached the maximum peak on 12 d and then decreased until 27 d. After 12 d, the MT-treated fruits showed significantly higher APX activity than the control, in which the mean values for the MT treatment were twice as high as the control treatment in ‘Guiqi’. On the contrary, the APX activity in ‘Tainong 1’ showed a continuous increase from 3 d to 24 d. MT treatment showed significantly higher APX activity (14.59 ± 0.13 U min^−1^ g^−1^ FW) than the control (13.82 ± 0.14 U min^−1^ g^−1^ FW) (*p* < 0.05). At the end of the storage period, the significantly higher APX between the MT treatment and the control was more pronounced in ‘Guiqi’ than in ‘Tainong 1’.

### 3.5. Effect of MT on Gene Relative Expression

#### 3.5.1. PAL and PPO Genes

**Figure 9:**
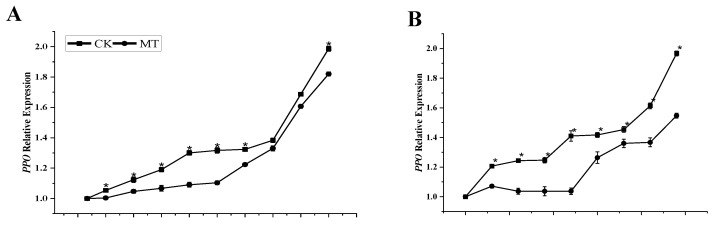

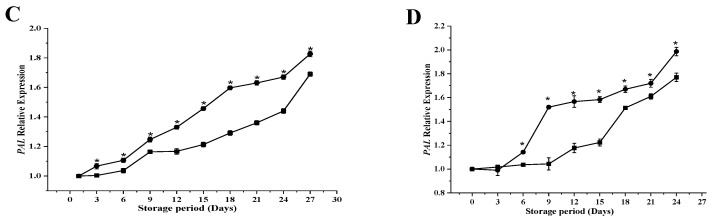
*PAL* relative gene expression (**A**,**B**) and *PPO* relative gene expression (**C**,**D**) of ‘Guiqi’ (**A**,**C**) and ‘Tainong 1’ (**B**,**D**) fruits treated with 1000 μmol L^−1^ MT. Each value is the mean for three replicates and vertical bars indicate the standard error (SE). Error bars with an asterisk (*) on the same storage period show a significant difference between the treatments (*p* < 0.05).

The expression of the *PAL* and *PPO* genes is depicted in Figure 9. The results showed that transcripts of these two genes in both the control fruits and the MT-treated fruits increased throughout the storage period. MT treatment upregulated the expression of the *PAL* gene (Figure 9A,B) and down-regulated the expression of the *PPO* gene (Figure 9C,D) during most stages of the storage. There was a statistically significant difference between the MT treatment and the control (*p* < 0.05) in the relative expression of both genes. The cultivars displayed slightly different gene expression profiles; ‘Tainong 1’ was more responsive to the *PPO* gene than ‘Guiqi’. Nevertheless, MT displays a typical pattern of browning-related gene expression in both cultivars.

#### 3.5.2. MnSOD and APX Genes

**Figure 10:**
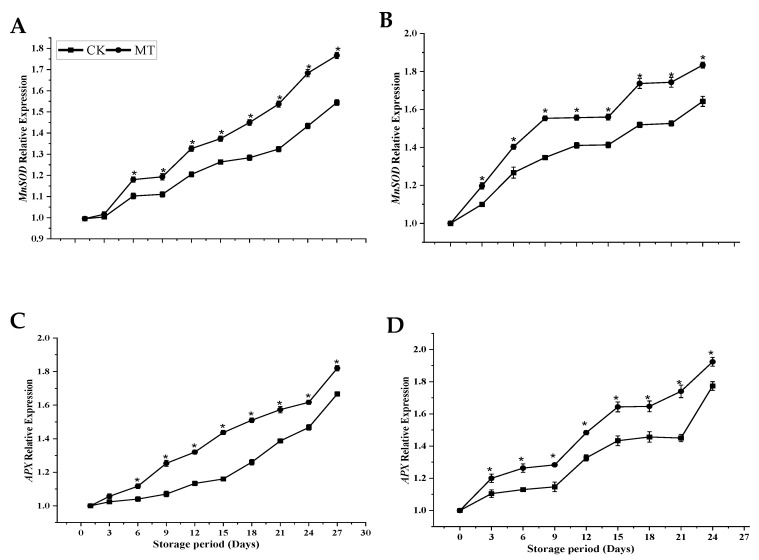
*MnSOD* relative gene expression (**A**,**B**) and *APX* relative gene expression (**C**,**D**) of ‘Guiqi’ (**A**,**C**) and ‘Tainong 1’ (**B**,**D**) fruits treated with 1000 μmol L^−1^ MT. Each value is the mean for three replicates, and vertical bars indicate the standard error (SE). Error bars with an asterisk (*) on the same storage period show a significant difference between the treatments (*p* < 0.05).

Figure 10 illustrated the effect of MT treatment on the relative gene expression level of antioxidant-related genes during cold storage. The results showed that MT treatment significantly upregulated the *MnSOD* genes (Figure 10A,B) and *APX* genes (Figure 10C,D) in both cultivars of mango during storage (*p* < 0.05) versus the control. Moreover, a slight difference in the gene expression profiles displayed by the two cultivars was observed but the difference was insignificant.

## 4. Discussion

Mango is a seasonal fruit; one of its primary constraints for continuous supply in the market is its climacteric nature. As a result, an attempt to minimize postharvest fruit deterioration, extend shelf life and maintain quality are critical in the supply chain of mango fruits. Concerning storage characteristics, our results showed that MT treatment delayed the increasing weight loss and slowed down the decreasing firmness of fruits compared to the control treatment in both cultivars (Figure 1A–D). Our results agree with previous research by Liu et al. and Bhardwaj et al. [15,16], confirming that MT treatment significantly delays fruit weight loss and firmness and the rate of respiration in mango fruits. Fruit weight loss and flesh firmness loss are two critical parameters that influence the storage of mango fruits due to their climacteric nature. Therefore, delaying fruit weight loss and firmness by MT treatment might result from the reduced activity of enzymes related to fruit softening and respiration, which was observed by Liu et al. [15].

These above results are supported by the fact that MT treatment significantly improves preservation via a mechanism involving the inhibition of respiration compared to the control in both cultivars (Figure 2C,D). However, the ‘Guiqi’ cultivar responded better to MT treatment concerning the delayed respiration rate than ‘Tainong 1’. In agreement with our results, the respiration rate in peach and pear fruits was significantly inhibited by 0.1 Mm MT under storage conditions [11,25]. It was also confirmed by Bhardwaj et al. [16] that the efficiency of MT on quality preservation at chilling temperatures was associated with delayed ethylene production and the respiration rate of four mango cultivars. Michailidis et al. [27] assume that an active interplay may exist among respiration, cold stress, and MT in sweet cherries due to high displays of respiratory activity and low-temperature storage, which may induce an uncoupling of the respiratory chain, which gives rise to ROS. As a result, the inhibition of the respiration rate by MT suggests that MT can minimize the accumulation of ROS, thereby minimizing mango fruit softening and decay [28]. MT treatment significantly delayed fruit decay compared to control fruits in the current study regardless of the cultivar (Figure 2A,B). This result could be due to the decreased activities of browning-related (PPO) enzymes by MT treatment in both cultivars. In agreement with our results, Zhang et al. and Agham et al. [28,29] found that MT treatment decreases the rate of decay incidence in litchi and strawberry fruits, respectively, compared to the control treatment. The decay of postharvest fruits is usually accompanied by pathogen infection, which leads to fruit deterioration and spoilage. MT contains antioxidants with immune-modulatory and anti-inflammatory effects, indicating that it can inhibit bacterial, viral, and parasitic infections [7].

TSS and TA are critical parameters related to fruit ripening. Sugars represent a fundamental component of fruit’s edible quality, predominantly conferring sweetness and significantly influencing consumer satisfaction. According to the present results, MT does not significantly affect the TSS and TA content (Figure 3A–D) in both cultivars studied. However, a continuous decline in TA is apparent and is considered an important marker of faster senescence. Because respiration utilizes organic acids as a substrate, this process tends to decrease TA [16]. The TSS:TA ratio is a strong indicator of ripeness while also influencing fruit taste and tends to increase in parallel to increasing TSS and decreasing TA significantly. The TSS:TA ratio was also not significantly lower in response to the MT treatment in ‘Tainong 1’, but it was higher in ‘Guiqi’ than in the control (Figure 3E,F), following a recent study [14]. MT’s inability to influence important quality parameters like TSS, TA, and the TSS:TA ratio could be due to hydrolytic changes and the conversion of starch into simple sugars [30]. Han et al. [30] reported that there is a high possibility that MT might modulate sugar metabolism, which indicates that a higher TSS by MT treatment might reflect MT’s effect on the loss of glucose and fructose, the main sugars found in many fruits. Moreover, Bhardwaj et al. and Miranda et al. [16,31] found that MT treatment significantly affects the TSS, TA, and TSS:TA ratio in three cultivars of mango fruits and sweet cherry, respectively during most stages of the storage period. However, more research is needed to understand the interaction between MT and sugar metabolism.

Oxidative damage can affect the quality of harvested fruit, which is a process of physiological deterioration [32]. This study determined the effects of MT treatment on the AsA content and total phenol and flavonoid content in two mango cultivars and how they influenced the postharvest quality of mango fruits and antioxidative activities. In this study, MT treatment significantly delayed the decreasing rate of AsA in both cultivars at the end of the storage period (Figure 4A,B). These results suggested that the exogenous application of MT could increase the ability of the mango fruits to resist oxidative damage and maintain fruit quality by activating the key enzyme activities and the increasing accumulation of antioxidants in the ascorbate-glutathione (AsA-GSH) cycle regardless of the cultivars under study. However, there was a slight variation in the cultivar response to MT treatment, but the difference was insignificant. The AsA-GSH cycle is an essential component in the elimination of ROS. APX is an essential enzyme in the AsA-GSH redox pathway, which plays a crucial role in promoting the regeneration of GSH and AsA, ensuring the normal functioning of metabolic pathways, and maintaining the balance of reduced substances in plants [33]. In our study, MT significantly increased APX activity compared to the control (Figure 8C,D), which corresponds to higher AsA content in both cultivars.

Mango fruit is rich in several other types of antioxidant phytochemicals, such as phenolic [34,35]. Therefore, the loss of astringency during mango fruit ripening is associated with decreased phenolic content [36]. In this study, a delayed decrease in the total phenol and flavonoid content was observed more in the MT-treated fruits (Figure 5A–D) than in the control, regardless of the cultivar, and the trend was similar in both cultivars during storage. Phenols play vital roles in maintaining the nutritional quality of fruits concerning bitterness, flavor, and stringency. Furthermore, phenolic compounds can protect membrane lipids from peroxidation by avoiding the occurrence and propagation of oxidative chain reactions [37]. Flavonoids, on the other hand, are potent antioxidants that play a crucial role in minimizing decay and disease in fruits. Both phenols and flavonoids are described as secondary metabolites that help in increasing the antioxidant characteristics of fruits via ROS scavenging activities [14]. Therefore, the higher content of total phenol and flavonoids by MT treatment at the end of the storage period indicates that MT plays a significant role in inhibiting postharvest fruit deterioration and minimizing qualitative loss in mango during storage. This result correlates with respiration inhibition by MT (Figure 2C,D) since an increase in respiration rate leads to an increase in the production of secondary metabolites.

To determine the physiological and molecular mechanism of the MT effect in two cultivars concerning fruit postharvest quality, an analysis of the enzyme activities related to browning and their relative gene expressions was determined during storage. MT treatment significantly delays the PPO enzyme activities compared to control fruits in the current study, regardless of the cultivar (Figure 7A,B). However, PPO activities in MT treatment were more pronounced in ‘Tainong 1’ than in ‘Guiqi’. In agreement with our results, the delayed increase in browning-related enzymes such as PPO in litchi fruits upon MT treatment was consistent with the rise in total phenol, flavonoid, and anthocyanin contents, which delayed the pericarp browning and maintained quality [38]. Studies have shown that membrane damage triggers a loss of subcellular compartmentalization, resulting in contact between browning related-enzymes (PPO) and phenolic substrates, consequently leading to enzymatic browning in harvested horticultural crops. In the current study, delayed increases in PPO activities (Figure 7A,B) concurrent with higher levels of total phenol and flavonoid contents were shown in MT-treated mango fruit, indicating that MT might inhibit enzyme-catalyzed phenolic oxidation and delay fruit peel browning, which is an indicator of decay in mango. It was also observed in this study that the rate of PPO inhibition by MT treatment was cultivar dependent since ‘Tainong 1’ inhibited PPO activity more than ‘Guiqi’.

The PAL enzyme, responsible for synthesizing polyphenols in plants, is located at a branch point between primary and secondary metabolism [39]. It enters secondary metabolism when the enzyme PAL catalyzes the elimination of ammonium, converting phenylalanine into cinnamic acid, a precursor of phenols such as flavonoids [39]. Our results showed a higher PAL activity in MT-treated fruits than in controls (Figure 7C,D) in both cultivars, suggesting that MT could minimize fruit decay by enhancing the defense-related secondary compounds that act against biotic and abiotic stress during storage. Furthermore, PAL has been demonstrated in the metabolic activity of many higher plants and is a crucial enzyme in synthesizing several defense-related secondary compounds like phenols and lignins [40]. In our study, the increase in the activity of PAL by MT treatment correlates to an increase in total phenol and flavonoid content, demonstrating that MT treatment effectively inhibited biotic and abiotic stresses, thereby minimizing fruit decay and elevating antioxidant processes during the cold storage of mango. However, the enhancement of the PAL enzyme was higher in ‘Tainong 1’ than in ‘Guiqi’ during the storage. In agreement with our results, the activity of the PAL enzyme was found to be increased by MT, which promotes the accumulation of total phenols and is beneficial to inhibiting fungal decay and prolonging the shelf-life of postharvest peach [13].

To shed more light on the activity of browning-related enzymes, we performed a qRT-PCR to determine the relative gene expression of the above enzymes. We found that MT treatment upregulated *PAL* genes and inhibited the *PPO* gene during storage (Figure 9A–D). The upregulation of the *PAL* gene and downregulation of the *PPO* gene correlate with their specific enzyme activities by MT treatment in this study.

ROS accumulation may damage lipids and form toxic products like MDA, indicating oxidative stress in plants. This phenomenon is known as lipid peroxidation and is triggered in part by lipoxygenase activity, resulting in cell membrane deterioration. Furthermore, our results showed that MT treatment minimized the MDA content (Figure 6A,B) at the end of the storage period compared to the control in both cultivars. However, the MT effect concerning the MDA content was more pronounced by ‘Guiqi’ than ‘Tainong 1’ at the end of the storage period. This result indicates that MT affects lipid peroxidation inhibition and minimizes oxidative stress in mango fruits during cold storage.

Plants have antioxidant systems which scavenge ROS and protect cells from ROS-induced injuries [40]. The enzymatic antioxidant system is also a primary way to control ROS production, which regulates the degree of lipid peroxidation [41]. SOD and APX are among the most important antioxidant enzymes for scavenging ROS. In our study, MT increased the activities of SOD and APX compared to the control-treated fruits (Figure 8A–D) in both cultivars, but the pattern of the MT effect was different between the two cultivars. This result indicates that MT could enhance the antioxidative processes to maintain fruit quality by improving ROS scavenging in mango fruits during cold storage. Exogenous MT treatment was also found to induce apple disease resistance by enhancing the activity of POD, SOD, and CAT and inducing the synthesis of endogenous MT [42].

Specific genes associated with antioxidant enzyme activities were unclear in mango and their roles in modulating fruit decay and maintaining quality. To understand the role of genes in the antioxidant system by MT treatment, the expression patterns of two genes were assessed in both mango cultivars during cold storage. The transcription levels of these genes (*MnSOD* and *APX*) in both cultivars were higher in MT-treated fruits than in control during storage (Figure 10A–D), but the magnitude of expression between the cultivars differs. Therefore, *MnSOD* and *APX* may be the significant genes regulating fruit’s antioxidant system. The high expression of antioxidant-related genes was essential for maintaining the storage quality of fruit by delaying fruit decay and enhancing the antioxidant system of mango fruits. It was reported that the upregulated expression of *SOD*, *CAT*, and *POD* might contribute to prolonging the shelf life of kiwifruit [43], which agrees with our results in this study. Another study discovered that MT upregulated genes coding for *CAT*, *APX*, *Mn-SOD*, and *Cu/Zn-SOD* in two cultivars of sweet cherry led to improved fruit quality during cold storage [31]. The results suggested that MT treatment might mitigate the fruit decay of mango, possibly by activating antioxidant enzymes and related genes.

**Figure 11:**
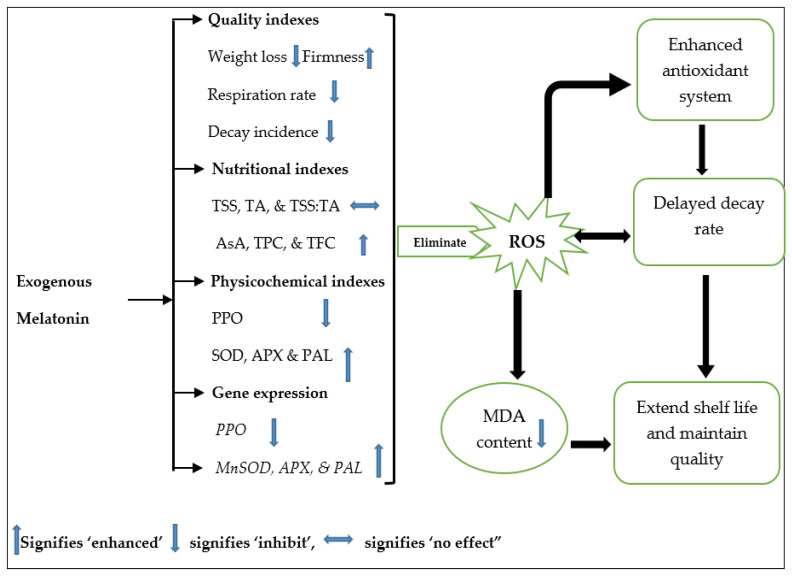
Schematic representation of melatonin (MT) antioxidant system enhancement. MT maintains the increase in weight loss, respiration rate, and decay and maintains the decrease in firmness. MT does not affect TSS, TA, and TS:TA. MT eliminates ROS directly and inhibits MDA content. It also increases the levels of PAL enzyme activity, enzymatic antioxidants (SOD, APX), and non-enzymatic antioxidants (AsA, TFC, TPC) and minimizes the activity of the PPO enzyme. MT enhanced the expression of genes related to the antioxidant system (*MnSOD* and *APX*) and the *PAL* gene and inhibited *PPO* relative gene expression. Abbreviations: TSS, total soluble solids; TA, titratable acidity; AsA, ascorbic acid content; TPC, total phenol content; TFC, total flavonoid content; PPO, polyphenol oxidase; SOD, superoxide dismutase; APX, ascorbate peroxidase; and PAL, phenylalanine ammonia-lyase.

To simplify the understanding of the mechanism of MT’s ability to delay mango fruit decay and enhanced the antioxidant system, the results of the experiment are summarize in the schematic diagram (Figure 11) including all the parameters assayed in this study. The diagram highlighted the pathway in which MT actions contributed to shelf life extension and maintaining quality in mango during cold storage.

## 5. Conclusions

In conclusion, it was observed that the storage behavior of the two cultivars differs slightly in response to MT treatment. ‘Guiqi’ lasted for a 27 d storage period while ‘Tainong 1’ lasted for 24 d. The study also discovered that MT maintained the quality of mango during storage by delaying weight loss, firmness, respiration rate, and decay incidence more than the control treatment in both mango cultivars. This result is evident with reduced PPO activity and enhanced PAL enzyme activity. However, MT did not affect TSS, TA, and the TSS:TA ratio in this study in both cultivars. Furthermore, MT was also found to delay the MDA content, while increasing SOD and APX enzyme activities, which correlates with maintaining a higher concentration of total phenols, flavonoid content, and AsA content during cold storage. The inhibition of the PPO enzyme and increase in the activities of the PAL, SOD, and APX enzymes by MT treatment correlates with the gene expressions. MT treatment was also cultivar dependent on specific parameters being studied. Therefore, this study could give a broader insight into the role of MT in the postharvest technology of mango fruits during storage.

## Figures and Tables

**Table 1:** The sequences of specific primers used for qRT-PCR quantification.

Gene Name	Gene ID	Forward Primer (5′-3′)	Reverse Primer (5′-3′)
*MnSOD*	123228203	TAACCGAATCCGTCCGCCTTGG	CCTCCGCCGTTGAACTTGATGG
*APX*	123203234	CTTCTTCTCTGTCGTTCTCT	TCTCGCACTCTTCAACTG
*PAL*	123193566	CTGGCTGGTATCAGTAGTG	CCTGGATGGTGCTTCAAT
*PPO*	123193265	TAGCACACGCAGCGGAGTTGAA	CCCAGTTGCCACCTCATCCTCA
*ACTIN*	123216838	AGACCACCTACAACTCCAT	ATCCTCCAATCCAGACACT

## Data Availability

The data are available upon request from the corresponding author.
